# Glioblastoma and Internal Carotid Artery Calcium Score: A Possible Novel Prognostic Partnership?

**DOI:** 10.3390/jcm13051512

**Published:** 2024-03-06

**Authors:** Francesco Pasqualetti, Michela Gabelloni, Lorenzo Faggioni, Giovanni Donato Aquaro, Fabrizio De Vietro, Vincenzo Mendola, Nicola Spina, Jessica Frey, Nicola Montemurro, Martina Cantarella, Mario Caccese, Giovanni Gadducci, Noemi Giannini, Silvia Valenti, Riccardo Morganti, Tamara Ius, Maria Caffo, Giuseppe Vergaro, Mirco Cosottini, Antonio Giuseppe Naccarato, Giuseppe Lombardi, Guido Bocci, Emanuele Neri, Fabiola Paiar

**Affiliations:** 1 Radiation Oncology Unit, Azienda Ospedaliero Universitaria Pisana, 56123 Pisa, Italy; martina.cantarella@ao-pisa.toscana.it (M.C.); ggadducci@gmail.com (G.G.); noemi.giannini@yahoo.com (N.G.); silvia.valenti83@gmail.com (S.V.); fabiola.paiar@unipi.it (F.P.); 2 Department of Surgical, Medical and Molecular Pathology and Critical Care Medicine, University of Pisa, 56126 Pisa, Italy; michela.gabelloni@gmail.com; 3 Academic Radiology, Department of Translational Research, University of Pisa, 56126 Pisa, Italy; lorenzo.faggioni@unipi.it (L.F.); giovanni.aquaro@unipi.it (G.D.A.); fabrizio.devietro@gmail.com (F.D.V.); mendolavincenzo.vm@gmail.com (V.M.); n.spina@studenti.unipi.it (N.S.); j.frey@studenti.unipi.it (J.F.); emanuele.neri@unipi.it (E.N.); 4 Department of Neurosurgery, Azienda Ospedaliero Universitaria Pisana, 56123 Pisa, Italy; nicola.montemurro@unipi.it; 5 Oncology Unit 1, Department of Oncology, Veneto Institute of Oncology IOV-IRCCS, 35128 Padua, Italy; mario.caccese@iov.veneto.it (M.C.); giuseppe.lombardi@iov.veneto.it (G.L.); 6 Section of Statistics, University Hospital of Pisa, 56124 Pisa, Italy; r.morganti@ao-pisa.toscana.it; 7 Neurosurgery Unit, Head-Neck and NeuroScience Department, University Hospital of Udine, 33100 Udine, Italy; tamara.ius@gmail.com; 8 Department of Neurosurgery, University of Messina, 98122 Messina, Italy; mariella.caffo@unime.it; 9 Interdisciplinary Center for Health Sciences, Scuola Superiore Sant’Anna, 56127 Pisa, Italy; vergaro@ftgm.it; 10 Cardiology Division, Fondazione Toscana Gabriele Monasterio, 56127 Pisa, Italy; 11 Department of Neuroradiology, University of Pisa, 56126 Pisa, Italy; mirco.cosottini@unipi.it; 12 Division of Pathology, Department of Translational Research and New Technologies in Medicine and Surgery, University of Pisa, 56126 Pisa, Italy; giuseppe.naccarato@unipi.it; 13 Department of Clinical and Experimental Medicine, Clinical Pharmacology, University of Pisa, 56126 Pisa, Italy

**Keywords:** glioblastoma, biomarkers, calcium score, radio-chemotherapy, geriatric patients, comorbidity

## Abstract

**Purpose:** Clinical evidence suggests an association between comorbidities and outcome in patients with glioblastoma (GBM). We hypothesised that the internal carotid artery (ICA) calcium score could represent a promising prognostic biomarker in a competing risk analysis in patients diagnosed with GBM. **Methods:** We validated the use of the ICA calcium score as a surrogate marker of the coronary calcium score in 32 patients with lung cancer. Subsequently, we assessed the impact of the ICA calcium score on overall survival in GBM patients treated with radio-chemotherapy. **Results:** We analysed 50 GBM patients. At the univariate analysis, methyl-guanine-methyltransferase gene (MGMT) promoter methylation (*p* = 0.048), gross total tumour resection (*p* = 0.017), and calcium score (*p* = 0.011) were significant prognostic predictors in patients with GBM. These three variables also maintained statistical significance in the multivariate analysis. **Conclusions:** the ICA calcium score could be a promising prognostic biomarker in GBM patients.

## 1. Introduction

Glioblastomas (GBMs) are the most common and aggressive primary malignant tumours of the central nervous system [1,2]. Despite significant advancements in the treatment of various extracranial solid tumours leading to improved survival rates, the prognosis for individuals afflicted by this deadly disease remains disappointingly grim. Over the past two decades, clinical research in the field of GBMs has witnessed limited progress since a ground-breaking study by Stupp et al. in 2005 [3]. Managing GBMs is hard because they are biologically complex and have traits like being very invasive, resistant to treatment and able to take advantage of the complex neural microenvironment. These characteristics contribute to the limited success of therapeutic interventions, making the development of innovative and effective treatment strategies an imperative yet elusive goal. The quest for improved outcomes in GBM patients continues to be a focal point of research, with a growing emphasis on unravelling the molecular intricacies that drive tumour progression. While advancements in understanding of the genetic and epigenetic landscapes of GBMs have expanded our knowledge, translating these insights into transformative clinical approaches remains a formidable challenge.

Novel therapeutic approaches, such as immunotherapy and targeted molecular interventions, hold promise for reshaping the treatment landscape for GBMs. However, the complicated relationship between the tumour and its surrounding environment, along with the fact that GBMs are very different from one another, makes it even more important to use all-encompassing and personalised strategies to fight this tough enemy. The ongoing pursuit of ground-breaking research endeavours aims to redefine the paradigm of GBM management, offering renewed hope for patients and clinicians alike [3,4].

Recent integrative studies have shed light on the variable prognoses of GBM patients, revealing the influence of several key factors. Age, the extent of resection (EOR), the size of necrosis, and some molecular markers [including the methylation status of O6-methylguanine-DNA methyltransferase (MGMT) and overexpression of the epidermal growth factor receptor (EGFR)] are all relevant factors accounting for the diversity of GBM outcomes. Understanding this intricacy could be crucial for developing targeted therapies and personalised treatment strategies [1,5,6]. Clinical evidence has shown that the incidence of GBM is higher in older individuals, who also tend to have a worse prognosis compared to younger ones, regardless of molecular and imaging characteristics [2,7,8]. Moreover, as suggested by some retrospective clinical studies, there could be some hidden, still unknown prognostic factors related to the presence of comorbidities in GBM patients that may affect their prognosis [9,10]. However, no biomarkers have been found so far revealing a correlation between the presence of comorbidities and prognosis in GBMs.

In the general population, a high coronary artery calcium score (CAC) is a long established significant risk factor for the development of acute coronary events [11]. Elevated calcium levels in the coronary arteries are a hallmark of atherosclerosis and are associated with an increased risk of myocardial infarction and stroke, the prevalence of which is notably higher in elderly patients [12].

In a retrospective study conducted in patients diagnosed with early-stage non-small-cell lung cancer undergoing computer tomography (CT) staging, Cuddy et al. found that 98% of patients with known atherosclerotic cardiovascular disease also had calcified coronary atherosclerotic plaques [13]. In the Multi-Ethnic Study of Atherosclerosis (MESA), which recruited 6814 participants and monitored them for a duration of more than 3 years, hazard ratios for major adverse cardiac events (MACEs), including myocardial infarction and coronary-related death, were 3.89 for a calcium score of 1–100, 7.08 for a score of 101–300, and 6.84 for a score greater than 300, respectively. Moreover, a direct correlation was found between a doubling of CACs and a 20% higher risk of MACEs [14].

The potential role of coronary artery calcification in stroke risk has also been suggested. In 2017, Chaikriangkrai et al. carried out a meta-analysis involving 13,262 asymptomatic individuals without evident cardiovascular disease [14], revealing that the collective risk ratio for incident stroke in patients with a CAC greater than zero compared to those with a CAC of zero was 2.95 (95% CI: 2.18–4.01, *p* < 0.001). The pooled incidence rates of stroke, as stratified by CAC, were as follows: 0.12%/year for CACs equal to 0, 0.26%/year for CACs between 1 and 99, 0.41%/year for CACs between 100 and 399, and 0.70%/year for CACs equal to or higher than 400. The authors concluded that the presence and severity of coronary artery calcification were linked to incident stroke during mid- to long-term follow-up.

Recently, several authors have conducted epidemiological studies on the association between cancer occurrence and the presence of atherosclerosis, intended as a biomarker of cardiac and cerebrovascular diseases. In a study by Li et al. (2022), involving 1600 patients stratified based on the presence of CAD or cancer, atherosclerotic coronary artery disease (CAD) and cancer were found to be independent risk factors for each other [15]. CAD prevalence was 78.9% in cancer patients compared to 52.4% in noncancer patients, and a multivariable logistic regression analysis showed that cancer patients had a significantly higher risk of developing CAD compared to those without cancer (odds ratio: 2.024, 95% confidence interval: 1.475 to 2.778, *p* < 0.001).

On the basis of these assumptions, our goal was to evaluate the impact of arterial calcifications as a prognostic factor in patients with GBM. Because the evaluation of coronary arteries is usually not included in the staging workup of GBM patients, we sought to measure the calcium score of the internal carotid arteries (ICAs) as a surrogate marker of the CAC. The purpose for which this study was conceived was not only to identify a prognostic factor capable of aiding in presurgical clinical decision making in patients with glioblastoma, but also to pave the way for the study of patient-related prognostic biomarkers.

## 2. Materials and Methods

### 2.1. Study Population

We retrospectively collected data from patients referred to the Radiotherapy Department of Pisa University Hospital from January 2010 to December 2019. Patients were enrolled according to the following criteria: (1) histological diagnosis of GBM; (2) age greater than 18 years; (3) Karnofsky performance score higher than 80; (4) postoperative treatment with radio-chemotherapy using temozolomide (75 mg/m^2^ during radiotherapy and 200 mg/m^2^ as a sequential regimen) [3,16]. Radiotherapy was delivered in the postoperative setting, 4–6 weeks after surgery, with a dose of 60 Gy in 30 sessions. The volume of interest was represented by the residual disease (assessed by postoperative MRI), the surgical bed, and a 2 cm margin.

Our primary endpoint was overall survival (OS), defined as the time interval from surgery to the date of death. Because treatment response and diagnosis of disease recurrence in some patients were assessed using either McDonald’s or RANO criteria, we decided not to use progression-free survival as a secondary endpoint [17]. Because of the retrospective nature of this study and the presence of potential bias related to incorrect clinical data collection, we preferred to avoid the analysis of potential comorbidities. All patients were evaluated and treated by a multidisciplinary team. Our local institutional review board approved this retrospective single-centre study (Comitato Etico di Area Vasta Nord Ovest [CEAVNO]; protocol 560/2015).

### 2.2. Validation of ICA Calcium Score as Surrogate Marker of CACs

Before evaluating patients with a GBM (on whom a CT examination limited to the brain was performed), 37 patients who had undergone chest and brain CT examinations (including precontrast scans) for baseline lung cancer staging were evaluated to seek a correlation between ICAs and coronary artery calcium scores. Chest and brain precontrast CT images with 2.5 mm slice thickness were transferred to a dedicated workstation, and for each patient, the Agatston scores were computed semiautomatically using the Calcium Scoring Plugin of the open-source software Horos Project™, version 3.0 (https://horosproject.org/ (accessed on 22 January 2024)). Five patients were excluded because of the close relationship between the calcified arterial wall and the bony skull base, preventing an accurate segmentation of ICA calcifications. The remaining 32 patients had a median ICA Agatston score of 46 (range: 0–1010) and a median coronary Agatston score of 12 (range: 0–572). A statistically significant correlation was found between ICA and coronary Agatston scores (*p* < 0.05).

### 2.3. Assessment of MR Images and MGMT Methylation of GBM Patients

Two neurosurgeons assessed the extent of surgery in terms of total gross tumour removal versus non-total gross tumour removal (i.e., partial resection or biopsy). The entire contrast-enhanced tumour was measured on contrast-enhanced T1-weighted sequences, whereas non-contrast-enhanced tumours were measured on FLAIR images. T2-weighted images were used whenever FLAIR images were unavailable. The methylation of the MGMT promoter was performed on the tumour specimen using the pyrosequencing technique.

### 2.4. Statistical Analysis

Categorical variables are reported as percentages, whereas continuous variables are reported as means ± standard deviations or medians and ranges as appropriate, according to the data distribution. The distributions of continuous variables were tested for normality using the Shapiro–Wilk test. The continuous CAC variable was transformed into quartiles and, subsequently, split in two categories (≤3, >3), where 3 represents the 25th percentile.

The overall survival (OS) curve was calculated using the Kaplan–Meier method, and the log-rank test was used to evaluate differences between curves. Univariate OS analysis was performed using the Cox regression, and all variables affecting survival (*p* < 0.1) were analysed together using a Cox multivariate model. The variables considered for univariate analysis were age, gender, Karnofsky performance status (KPS), MGMT methylation, and extent of surgery. The results of the Cox regression analysis were expressed in terms of hazard ratios (HRs), along with their 95% confidence intervals (95% CI) and *p*-values.

A *p*-value of 0.05 was set as the threshold of statistical significance. All analyses were carried out using SPSS version 28 (https://www.ibm.com/products/spss-statistics (accessed on 22 January 2024)).

## 3. Results

**Figure 1:**
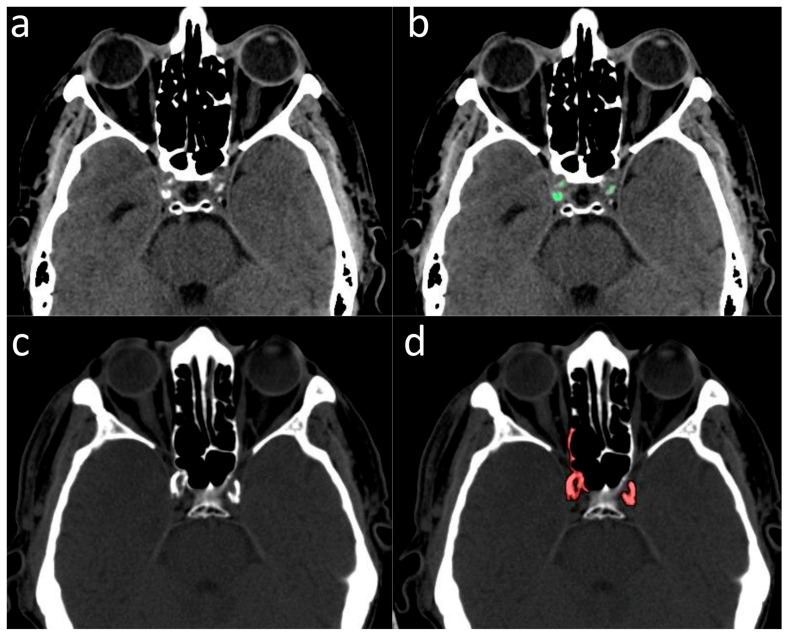
(**a**,**b**) Example of patient in whom the measurement of the ICA calcium score was feasible: (**a**) without calcium segmentation; (**b**) with calcium segmentation (highlighted in green). (**c**,**d**) Example of a patient in whom the measurement of ICA calcium score was not feasible due to overlap with the adjacent bony skull base: (**c**) without calcium segmentation; (**d**) with calcium segmentation (highlighted in red).

Data analysis was performed in August 2023. One hundred and ninety-six patients with GBM who met the enrolment criteria were identified from the Radiotherapy Department of Pisa University Hospital dataset. The ICA calcium scores were computed from brain CT images of GBM patients following the same procedure used for preliminary calcium score assessment in patients with lung cancer. Of them, 146 patients (74.5%) were excluded because of ICA proximity to the bony skull base, hindering an accurate quantification of ICA wall calcifications (Figure 1). Fifty patients (28 male, 22 female) were eligible for the final analysis.

Table 1 lists the features of the GBM patients included in the study. The ICA calcium scores at the 25th, 50th, and 75th percentiles were 3, 8.5, and 34, respectively. Sixteen patients had an ICA calcium score equal to or lower than three (patients in the first quartile), and thirty-four had a value higher than three (those in the other three quartiles). The ICA calcium score levels did not show a statistically significant correlation with patient age (*p* = 0.186).

The study cohort included patients with an average age of 63 years (range: 36–84). Methylation status of the MGMT gene promoter was assessed in 39 patients, revealing methylation in 28 patients and no methylation in 11 patients. The extent of surgical resection was evaluated in 32 patients, with 11 undergoing gross total resection and 21 opting for subtotal resection or biopsy.

**Figure 2:**
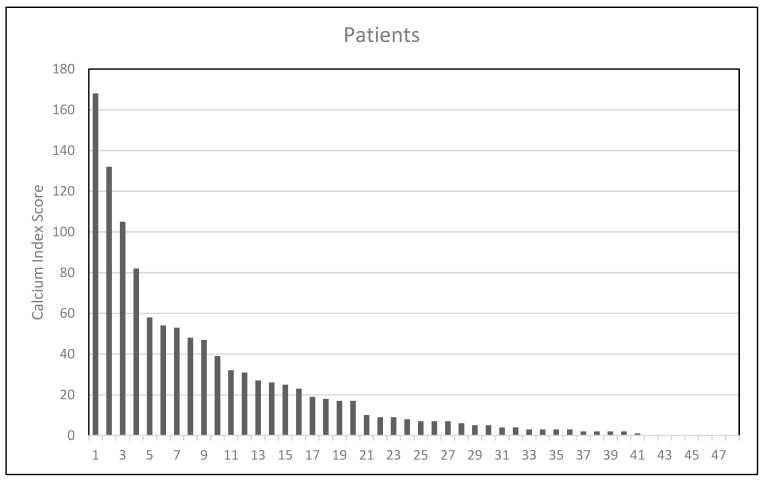
Distribution of ICA calcium score values.

The mean ICA calcium score was 32. The average patient OS was 17 months (95% CI: 13–21). A notable observation was the age distribution across quartiles, with an average of 59 years for the lowest quartile and 64 years for the other three quartiles (*p* = 0.087) (Figure 2).

**Figure 3:**
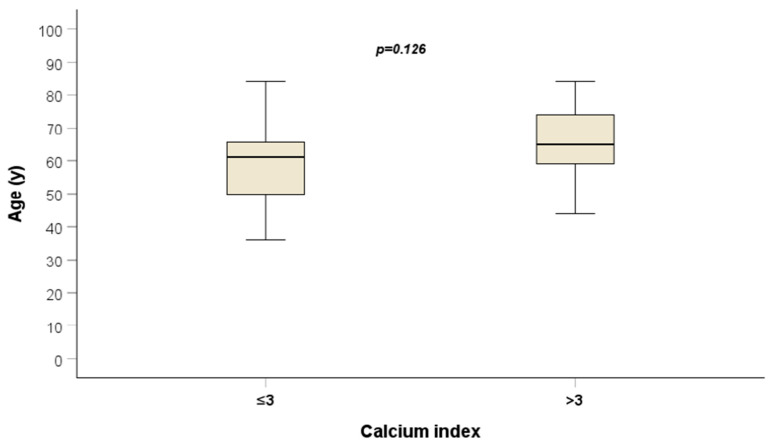
Correlation between patient age and ICA calcium levels.

**Figure 4:**
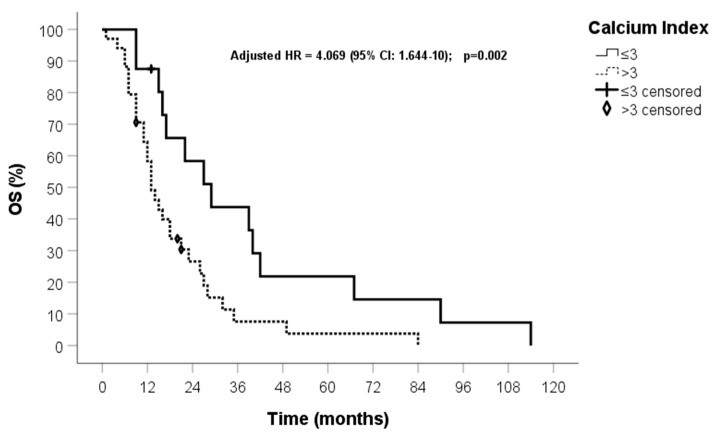
OS of patients grouped in the first quartile and the other three quartiles.

In the univariate analysis, MGMT promoter methylation, surgical radicality, and calcium score were predictors of OS (*p* < 0.05). These three variables maintained statistical significance in the multivariate analysis (Table 2). After patient stratification into quartiles, OS of 29 months (95% CI: 16–41) and 13 months (95% CI: 10–16) were found for patients in the first quartile and in the other three quartiles, respectively (HR 2.443, 95% CI: 1.229–4.855, *p* = 0.011) (Figure 3 and Figure 4). All patient deaths were directly correlated with GBM progression rather than with the presence of comorbidities.

## 4. Discussion

There are some limits to using surgical specimens to identify and develop biomarkers linked to prognosis or response to radio-chemotherapeutic treatments in patients diagnosed with glioblastoma. The outcomes and lengths of response following radio-chemotherapy treatments were not the same for patients whose histological characterisation showed the same markers, such as the mutation of the IDH1/2 gene and the methylation of the MGMT promoter. Certain factors influencing the aggressiveness of gliomas may not be present in the tumour sample but in the patients’ blood and may be related to certain clinical conditions of the patients. Most importantly, the study of tumour specimens has not produced markers that can be used to tailor postoperative treatment for patients with this terrible tumour. Following the diagnosis of a grade 4 glioma, the postoperative therapeutic approach is primarily determined based on the patients’ overall conditions and their age [8,16]. Furthermore, despite the results of various translational studies, there are no biomarkers that can enable stratification of patients based on radiosensitivity and factors correlated with better or worse response to radiotherapy [18,19].

The already dismal prognosis of GBM worsens with age and the presence of comorbidities at the time of diagnosis [7,10]. In this retrospective study involving 50 GBMs, we evaluated OS by stratifying patients based on clinical, radiological, and molecular factors. Overall, the main clinical results of our investigation are consistent with those of the existing literature, including an average OS of 17 months [20,21]. In addition, all our patients received the same postoperative treatment, had a Karnofsky performance status score greater than 80, and all deaths were solely correlated with the progression of GBM.

The arterial calcific burden has emerged as a valuable diagnostic tool in cardiovascular health assessment [22]. This kind of information is usually obtained by measuring quantitative parameters (such as CACs) from medical images and can provide crucial insights into the presence and extent of arterial calcification. Coronary calcification is a notable marker of atherosclerosis, a condition associated with the buildup of plaques that can lead to ischemic events. Understanding the role of arterial calcification is pivotal for risk assessment, early disease detection, and the implementation of preventive measures to maintain cardiovascular health. Research has demonstrated a strong correlation between elevated CACs and the risk of cardiovascular events, including myocardial infarction and stroke [23].

As a preliminary step of the present study, we decided to measure CACs in patients with lung cancer and verify that there was a sufficient correlation with the calcium score measured at internal carotid arteries. This step was necessary because chest CT for staging purposes is usually not performed in GBM patients. At the planning stage of the study, we were aware that this step could limit the significance of our results. While there is clinical evidence regarding the association between CACs and the presence of cardiovascular and cerebrovascular diseases, to our knowledge, there are no studies evaluating the impact of calcium scores measured based on the carotid arteries. On the basis of the further analysis of the MESA data, Gepner et al. found that the CACs yielded superior predictive accuracy, discrimination, and reclassification for cardiovascular disease and coronary heart disease compared to carotid ultrasound measurements [24]. However, when it comes to predicting and discriminating for stroke/transient ischemic attack, the performance of the two calcium scores was similar. It should be noted that the evaluation of calcium in carotid arteries was performed by Gepner at al. using ultrasound instead of CT. More encouraging data corroborating our findings were achieved by Shenouda et al. in 2021, who reported the results of a retrospective study carried out on 63 patients with unstable angina or positive stress test for myocardial ischaemia [25]. In these patients, the overall CACs showed a significant correlation with total carotid score (rho = 0.34, *p* = 0.007), particularly with the left carotid score (rho = 0.38, *p* = 0.002), while the correlation with the right carotid score was not statistically significant. Total CACs were related to both the left carotid bifurcation score (rho = 0.34, *p* = 0.008) and the proximal internal branch score (rho = 0.29, *p* = 0.026) in patients with severe coronary calcification. However, in patients with extensive coronary calcifications, no statistically significant association was found between total CACs and the left carotid bifurcation score or the proximal internal branch score. It should also be pointed out that this analysis was performed in patients symptomatic for cardiovascular disease, therefore introducing a selection bias [26].

In our study, the ICA calcium score seems to hold prognostic potential on both univariate and multivariate analyses, with a longer survival occurring in patients with a lower calcium score compared to those with a higher calcium score (*p* = 0.011). Intriguingly, age did not prove to be a prognostic factor and showed no correlation with calcium score. The average age of patients in the lowest quartile was 59 years, while it was 64 years in the other three quartiles (*p* = 0.087). Overall, these findings suggest that the ICA calcium score might find its role in the clinical management of patients with GBM as a potential predictor of patient survival, thus aiding risk stratification and personalised treatment.

Despite a growing number of investigations exploring the relationship between age, comorbidities, and the prognosis of GBM patients, to our knowledge this is the first investigation assessing the relationship between the CAC and life expectancy of patients affected by this deadly disease. In 2019, Villani et al. reported the results of a retrospective study carried out on 118 elderly patients with GBM [27], showing that in GBM patients with the same histological diagnosis, high cumulative illness rating scale (CIRS), comorbidity index, and performance status played a predictive role in survival. In 2020, Ius et al. conducted a large retrospective multicentre study on 332 elderly patients who had undergone surgery for GBM, showing that OS was affected by age and providing a new clinical prognostic survival score [28]. In 2019, Carr et al. reported the results of a retrospective study of 163 GBM patients treated between 2005 and 2015 [9], showing that in a multivariate analysis, a history of asthma (HR 2.22, 95% CI: 1.02–4.83, *p* = 0.04) and hypercholesterolemia (HR: 1.99; 95% CI: 1.11–3.56; *p* = 0.02) were correlated with reduced survival rates. In contrast, Barz et al. evaluated the impact of the age-adjusted Charlson comorbidity index (ACCI) in patients diagnosed with GBM, finding no association between ACCI and prognosis [10]. However, in that study, the patient sample was heterogeneous and not all GBMs had been administered in the same postoperative treatment, with only 27 out of 123 patients having received postoperative radiotherapy [26].

Our findings lay the groundwork for subsequent investigations seeking to validate our understanding of the potential influence of comorbidities on prognosis in patients with glioblastoma. Notably, individuals in the first quartile of our cohort, exhibiting better survival outcomes in both univariate and multivariate analyses, displayed internal carotid artery (ICA) calcium scores lower than 3. This suggests minimal calcification and, consequently, a reduced probability of plaque formation associated with atherosclerosis. This, in turn, correlates with an overall diminished likelihood of cardiovascular and cerebrovascular events. These insights underscore the intricate interconnections between the tumour microenvironment, comorbidities, and prognostic outcomes in GBM patients, paving the way for more nuanced and personalised therapeutic approaches.

Although our results seem to be promising, our study has limitations that make it a proof of concept to be confirmed through larger and prospective studies. The most significant limitation is represented by the small number of cases evaluated. Moreover, the measurement of the CACs was limited by not being routinely applicable to all patients with GBMs. In the preliminary phase of assessing the feasibility of the ICA calcium score as a surrogate of CACs, we observed that the mean ICA calcium score in lung cancer patients was 44, while the same value in GBM patients was 32. Furthermore, in 32 out of 37 (86.5%) lung cancer patients it was possible to measure the calcium score of the carotid arteries, whereas this was feasible only in 50 out of 196 GBM patients (25.5%). These differences could be correlated with the different risk factors for the onset of these two tumours. For example, in patients with lung cancer, a smoking habit is a known risk factor for the presence of calcium in the arteries, as well as one of the most common etiological agents. Moreover, we measured the ICA calcium score in terms of the Agatston score as a surrogate of coronary CACs, although to our knowledge, currently there are no established dedicated protocols for quantifying ICA calcifications with CT. Finally, it would have been interesting to correlate the presence and severity of atherosclerosis-related comorbidities with calcium score and prognosis. Unfortunately, data regarding the nononcological history of some patients may have been omitted because of the retrospective nature of this study, and including this information would have implied the risk of a selection bias.

In addition to providing a potential prognostic biomarker, our findings can serve as a starting point for future translational studies aimed at understanding how the presence of comorbidities in GBM patients may affect the biological aggressiveness of this tumour. If confirmed through prospective studies with a larger sample size, our results could suggest that some patient-related factors, other than tumour-related ones, might play a prognostic and possibly predictive role. An explanation for the relationship between the presence of cardiovascular and cerebrovascular comorbidities (related to a high calcium score) and the prognosis of GBM patients could lie in the action of miRNAs contained in exosomes due to the formation of atherosclerotic plaques, stroke, or myocardial infarction [29]. Despite having opposite outcomes, some exosomal miRNAs share the same biological function in the context of an atherosclerotic plaque or a tumour, such as exosomal miR-126, which plays a significant role in tissue recovery following a stroke by attenuating its progression [30]. However, preclinical studies have also shown that through the same gene target, miR-126 can be involved in the progression and invasion of various solid tumours. Additionally, exosomes released by atherosclerotic plaques have shown the upregulation of miRNA-21 [31]. An increased expression of this mi-RNA is, among other functions, associated with the stimulation of inflammatory response within the vascular wall, macrophage activation, and increased cell survival inside atherosclerotic plaques [32]. Nevertheless, exosome miRNA-21 is also overexpressed in glioblastomas, in which it plays a significant role in tumour development and progression. Upregulation of this miRNA is linked to faster growth and invasion and higher resistance to treatments and immunomodulation, making the immune system less active in tumour surveillance and suppression [33,34].

## 5. Conclusions

On the basis of our findings, the ICA calcium score might represent a useful prognostic biomarker in GBM patients. Future prospective studies with a larger sample size will be required to validate our results and to establish the value of the ICA calcium score as a prognostic factor in GBM patients. Translational studies will also be needed to investigate the association between CACs and unfavourable prognosis of patients with GBM.

## Figures and Tables

**Table 1:** Patient characteristics.

Characteristic	Statistics
**Gender**	
M	56%
F	44%
**Median Age (years)**	62 (range: 36–84)
**MGMT methylation**	
no	28.2%
yes	71.8%
**Extent of surgery**	
GTR	36.4%
STR	63.6%
**ICA calcium score expressed in quartiles**	
25th	3
50th	8.5
75th	34
**Categorical ICA calcium score**	
**≤3**	16 (32%)
**>3**	34 (68%)
**OS median time (months)**	17 (95% CI: 13.1–20.9)
**PFS median time (months)**	9 (95% CI: 6.6–11.4)

MGMT: methyl-guanine-methyltransferase; GTR: gross tumour removal; STR: subtotal tumour removal; OS: overall survival; PFS: progression-free survival.

**Table 2:** Results of univariate and multivariate analyses.

End Point: OS	Univariate Analysis			Multivariate Analysis		
Factor	RC	HR (95% CI)	*p*-Value	RC	HR (95% CI)	*p*-Value
**Age**	0.019	1.019 (0.991–1.048)	0.186			
**Gender** **(0) M, (1) F**	−0.282	0.754 (0.405–1.404)	0.373			
**Calcium index** **(0) ≤3 (1) >3**	0.893	2.443 (1.229–4.855)	0.011	1.912	6.767 (2.190–21)	0.001
**MGMT** **(0) no, (1) yes**	−0.859	0.423 (0.181–0.992)	0.048	−2.712	0.066 (0.009–0.472)	0.007
**Extent of surgery** **(0) GTR, (1) STR**	0.978	2.659 (1.188–5.949)	0.017	1.475	4.369 (1.520–12.6)	0.006

RC: regression coefficient; HR: hazard ratio; GTR: gross tumour removal; STR: subtotal tumour removal.

## Data Availability

The data presented in this study are available on request from the corresponding author.
